# The *Caenorhabditis elegans* and *Haemonchus contortus* beta-tubulin genes cannot substitute for loss of the *Saccharomyces cerevisiae* beta-tubulin gene

**DOI:** 10.17912/micropub.biology.000411

**Published:** 2021-06-30

**Authors:** Sophia B Gibson, Clare S Harper, Laura L Lackner, Erik C Andersen

**Affiliations:** 1 Molecular Biosciences, Northwestern University, Evanston, IL, 60208, USA; 2 Interdisciplinary Biological Sciences Program, Northwestern University, Evanston, IL, 60208, USA

## Abstract

To better understand the mechanism of resistance caused by putative interactions between beta-tubulin and benzimidazole compounds, we sought to purify nematode-specific beta-tubulins using heterologous expression after replacement of the single *Saccharomyces cerevisiae *beta-tubulin gene. However, we found that haploid yeast cells containing nematode-specific beta-tubulin genes were not viable, suggesting that nematode beta-tubulin cannot substitute for the loss of the yeast beta-tubulin gene.

**Figure 1. Caenorhabditis elegans and Haemonchus contortus beta-tubulin genes cannot substitute for loss of the sole Saccharomyces cerevisiae beta-tubulin TUB2:**
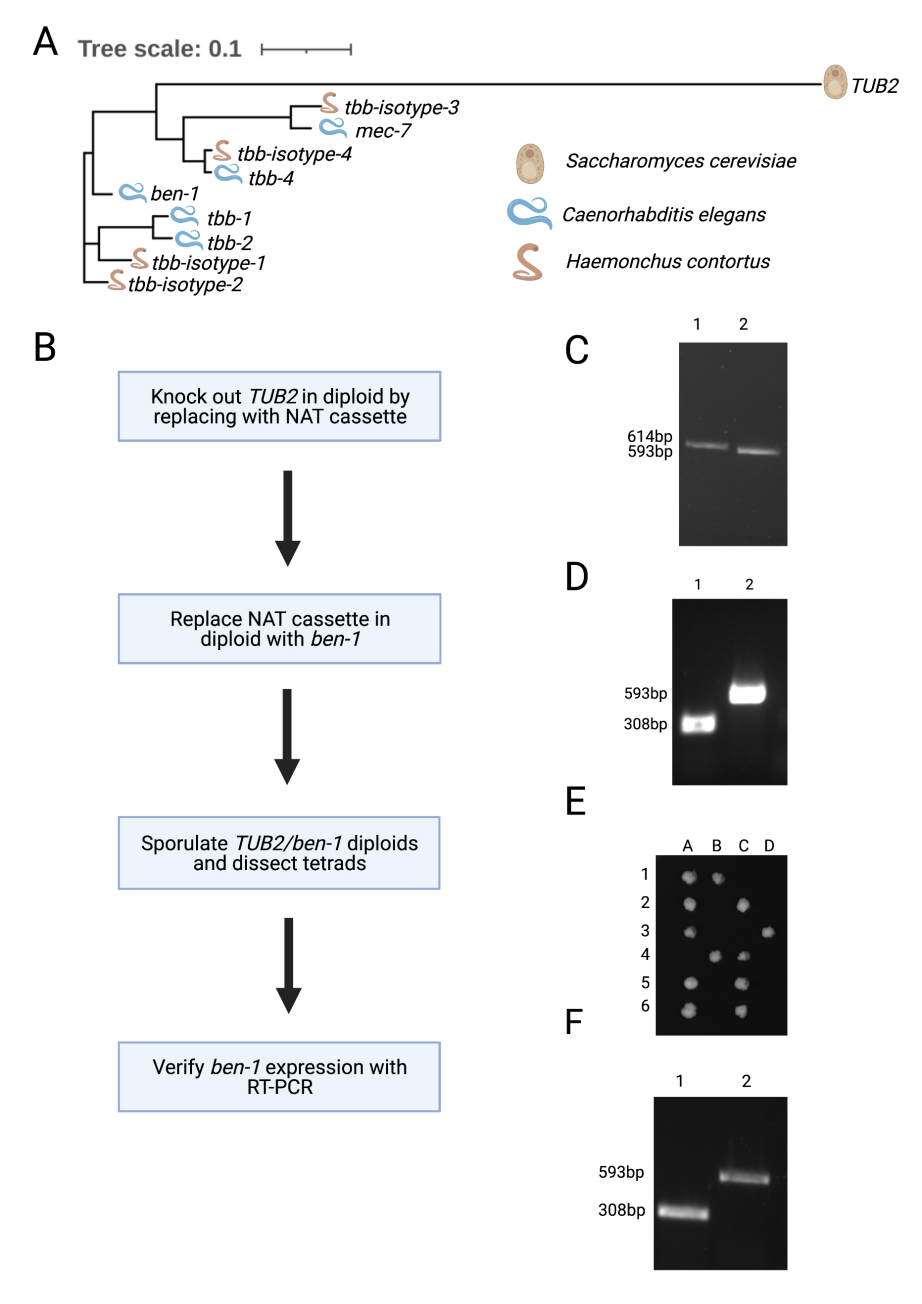
**A)** A phylogenetic tree of the beta-tubulin orthogroup for *C. elegans*, *H. contortus*,and *S. cerevisiae* is shown. The tree was created using MAFFT (Katoh *et al.*., 2002; Katoh & Standley, 2013) and IQ-TREE with ultrafast bootstrapping (Hoang *et al.*, 2018; Minh *et al.*, 2020). The tree was constructed using a LG+R3 substitution model selected by the highest Bayesian Information Criterion score using ModelFinder (Kalyaanamoorthy *et al.*, 2017). Visualization was performed using iTOL (Letunic & Bork, 2021). **B)** The experimental design for replacement of *S. cerevisiae TUB2* with *ben-1* is shown. For C through F, PCR products were separated on a 1% agarose gel with the sizes of each band shown on the left. **C)** Colony PCR confirmed replacement of *TUB2* with a NAT cassette. Lane 1 contained a fragment amplified from one primer downstream of *TUB2* and another internal to the NAT cassette. Lane 2 is a product amplified from a sequence internal to the MAT-alpha locus, which serves as a positive control. **D)** Colony PCR confirmed codon-optimized *ben-1* replacement at the *TUB2* locus. Lane 1 corresponded to primers for an internal *ben-1* fragment. Lane 2 corresponded to primers for an internal MAT-alpha sequence. **E)** Tetrad dissection results of *ben-1* replacement diploids are shown. Six tetrads (rows 1-6) were separated into four haploid cells each (lanes A-D). **F)** RT-PCR results confirmed *ben-1* expression in *S. cerevisiae*. An endpoint PCR product was amplified from cDNA reverse transcribed from RNA extracted from the *ben-1* replacement sample. Lane 1 corresponded to PCR products generated using primers for an internal *ben-1* fragment. Lane 2 corresponded to PCR products generated using primers for an internal MAT-alpha sequence. This figure was created using BioRender.com.

## Description

Parasitic nematode infections continue to have an enormous impact on human and livestock health worldwide (Hotez **et al.**, 2014; Kaplan & Vidyashankar, 2012). A limited arsenal of anthelmintic drugs are available to combat these infections. One of the most widely used classes is benzimidazoles (BZ), and resistance against this class is widespread (Kaplan & Vidyashankar, 2012). Previous studies to understand parasitic nematode resistance using the free-living model organism *Caenorhabditis elegans* showed that variation in the *C. elegans* beta-tubulin gene *ben-1*, an ortholog of beta-tubulins in parasitic nematodes, confers resistance to BZ drugs (Dilks **et al.**, 2020; Driscoll **et al.**, 1989; Hahnel **et al.**, 2018). The most common missense mutation resistance alleles are F167Y, E198A, and F200Y (Avramenko **et al.**, 2019; Mohammedsalih **et al.**, 2020). Although computational models have predicted that these amino acids are involved in the binding of BZ compounds to beta-tubulins, the binding remains to be investigated empirically at the structural level because nematode-specific beta-tubulin structures have not been created (Aguayo-Ortiz **et al.**, 2013; Hahnel **et al.**, 2018). To better understand the mechanisms of resistance, we sought to obtain those crystallographic structures.

To isolate BEN-1 for crystallographic studies, tags would permit the purification of this beta-tubulin from the other five beta-tubulins in *C. elegans*. Unfortunately, endogenous tags of BEN-1 eliminate its function (Dilks and Andersen, unpublished results). Because of this limitation, we turned to expression of three different versions of nematode beta-tubulins, *C. elegans* BEN-1 and two beta-tubulin isotypes from the parasitic nematode *Haemonchus contortus,* in *Saccharomyces cerevisiae*,which has a single beta-tubulin gene (Figure 1A)*.*

We used the following procedure to replace the *S. cerevisiae* beta-tubulin gene with each of the nematode beta-tubulin genes (Figure 1B). These replaced beta-tubulin genes were expressed using the native *S. cerevisiae* promoter. First, *TUB2* was deleted in diploid BY4743 cellsand replaced with the Nourseothricin N-acetyl transferase (NAT) cassette selection marker (Hansen **et al.**, 2003). Successful deletion of one copy of the *TUB2* coding sequence was verified using colony PCR of a sample that grew in the presence of NAT (Figure 1C). The NAT cassette was then substituted with yeast codon-optimized *C. elegans ben-1* along with the *HIS3* selection marker (Burke & Gould, 1994). Successful replacement of *ben-1* at the *TUB2* locus was verified using colony PCR on samples that grew in histidine-deficient conditions (Figure 1D). We sporulated diploids of the *ben-1* replacement strain then dissected tetrads to identify haploid cells that contained the *C. elegans* beta-tubulin gene *ben-1*. We found that dissected tetrads gave rise to only two viable haploid cells instead of four when grown on complete media (YPD) (Figure 1E). The same result was obtained after replacement of *TUB2* with either of the two *H. contortus* beta-tubulin isotype genes (data not shown). The two surviving colonies were also unable to grow in histidine-deficient conditions, suggesting that replacement of *TUB2* with nematode beta-tubulin genes is lethal. Using RT-PCR of the heterozygous diploid strain with one copy of *TUB2* and one copy of *ben-1* at the *TUB2* locus, we found that *ben-1* mRNA was expressed (Figure 1F), suggesting that the nematode beta-tubulin is deficient at protein expression, lacks proper stability, and/or cannot function with the yeast alpha-tubulin gene. Our results indicate that nematode beta-tubulin genes can not substitute for the yeast beta-tubulin gene *TUB2*.

Beyond the phylogenetic difference between nematode and yeast beta-tubulins, it has been observed that the dynamics of microtubules in *C. elegans* greatly differ from other eukaryotes (Chaaban **et al.**, 2018), suggesting that nematode beta-tubulins might be unsuitable replacements for yeast beta-tubulins. It is possible that both alpha- and beta-tubulin might need to be replaced by their nematode-specific versions to obtain viable yeast and allow for the characterization of benzimidazole and beta-tubulin binding to better understand the mechanism of resistance.

## Methods

**Strain Construction**

S288C derivative strains BY4741 and BY4742 (Brachmann **et al.**, 1998) were mated to make diploid BY4743.

**Plasmid Construction**

*pFA6-NAT* and *pKT128*

The construction for the plasmids containing the NAT cassette, pFA6-NAT, and the *S. pombe HIS3* marker, pKT128, have been outlined previously (Janke **et al.**, 2004; Sheff & Thorn, 2004).

*pECA101, pECA103, and pECA103*

Yeast codon optimized sequence for *C. elegans ben-1* and *H. contortus tbb-isotype-1* were cloned into a pUC57 vector and *H. contortus tbb-isotype-2* was cloned into a pJET1.2 vector by GenScript (Piscataway, NJ) to make pECA101, pECA102, and pECA103 respectively. The plasmid was cloned into DH-alpha competent cells and prepped with a QIAprep Spin Miniprep Kit (Qiagen, Hilden, Germany).

**Yeast Transformation**

Cells were grown overnight in YPD at 24°C, diluted to an OD of 0.15 and then grown to an OD of 0.5-1.0. Cells were resuspended in 100 mM Lithium acetate and combined with 50% PEG, 1M Lithium acetate, 10 mg/mL salmon sperm DNA, water, and the desired amplicon for integration. After incubating at 30°C for 30 minutes, DMSO was added and then reactions were heat shocked at 42°C for 15 minutes. Cells were washed and either recovered overnight in YPD and plated on YPD with NAT for NAT selection or resuspended in water and plated immediately on -HIS plates for *HIS3* selection. Plates were incubated for 48 hours at 30°C.

***TUB2* Knock-out**

A NAT cassette sequence flanked by the beginning and end of the *S. cerevisiae TUB2* sequence was amplified with *pFA6-NAT* and the following oligonucleotides (IDT, Coralville, IA)

oECA1756: ATGAGAGAAATCATTCATATCTCGACAGGTCAGTGTGGTACGGATCCCCGGGTTAATTAA

oECA1757: TTATTCAAAATTCTCAGTGATTGGTTCATCTTGGTTTTGTGAATTCGAGCTCGTTTAAAC

Oligonucleotides to verify NAT cassette replacement (IDT, Coralville, IA)

oECA1758: AACTGGTGCACTTAATCGCTG

oECA1759: CAATTCAACGCGTCTGTGAGG

Oligonucleotides for mating type positive control (IDT, Coralville, IA)

MAT-F: TTACTCACAGTTTGGCTCCGGTGT

MAT-R: GAACCGCATGGGCAGTTTACCTTT

***Ben-1* replacement**

A codon-optimized *C. elegans ben-1* sequence flanked by sequence upstream and downstream of *TUB2* was amplified with *pECA101* and the following oligonucleotides (IDT, Coralville, IA)

oECA1762: CTACTACAACTACAAAAGCAAAATCTCCACAAAGTAATATAATGAGAGAAATTGTTCATG

oECA1763: CATAAGAAATTCGCTTATTTAGAAGTGGCGCGCCTTATTCAGCGTCACCATC

An *S. pombe HIS3* selective marker was amplified with *pKT128* and the following oligonucleotides (IDT, Coralville, IA)

oECA1764: GATGGTGACGCTGAATAAGGCGCGCCACTTCTAAATAAGCGAATTTCTTATG

oECA1765: AGAGAAGAAGAAAGGTAAGAAAAAGAAAGGAAAGCAACTTAATCGATGAATTCGAGCTCG

Oligonucleotides to confirm the substitution or expression of *ben-1* (IDT, Coralville, IA)

oECA1769: CATACAATGCTACATTGTCAG

oECA1770: CAAAGCTCTATATGCTTGAG

**Sporulation and Tetrad dissection**

Cells were grown overnight in YPD at 24°C and then nitrogen starved in SPO Media at 24°C for 5-6 days until sporulated. Cells were then digested in 0.5 mg/mL zymolyase, resuspended in 1.2M sorbitol, and tetrads were dissected onto YPD plates and incubated for 48 hours at 30°C. Grown colonies were then struck on to -HIS plates and incubated overnight at 30°C.

**RNA extraction and RT-PCR**

Cells with *ben-1* substitution were grown to an OD600 of 10 overnight in YPD at 24°C and then pelleted and resuspended in High Salt RNA Buffer (0.3 M NaCl 20 mM Tris pH 8 10 mM EDTA 1% SDS), then separated with TE-saturated Phenol:Chloroform:Isoamyl alcohol. RNA was then extracted with Chloroform, precipitated with ethanol, and resuspended in water.

RNA was converted to cDNA using the iScript cDNA Synthesis kit (Bio-Rad, Hercules, CA). The *ben-1* verification and mating-type sequences were then amplified.
